# Supplementary figures and images for: Megacities as drivers of national outbreaks: The 2017 chikungunya outbreak in Dhaka, Bangladesh
Source: PLoS Negl Trop Dis. 2021 Feb 2;15(2):e0009106. doi: 10.1371/journal.pntd.0009106 (PMC7880496; doi:10.1371/journal.pntd.0009106)

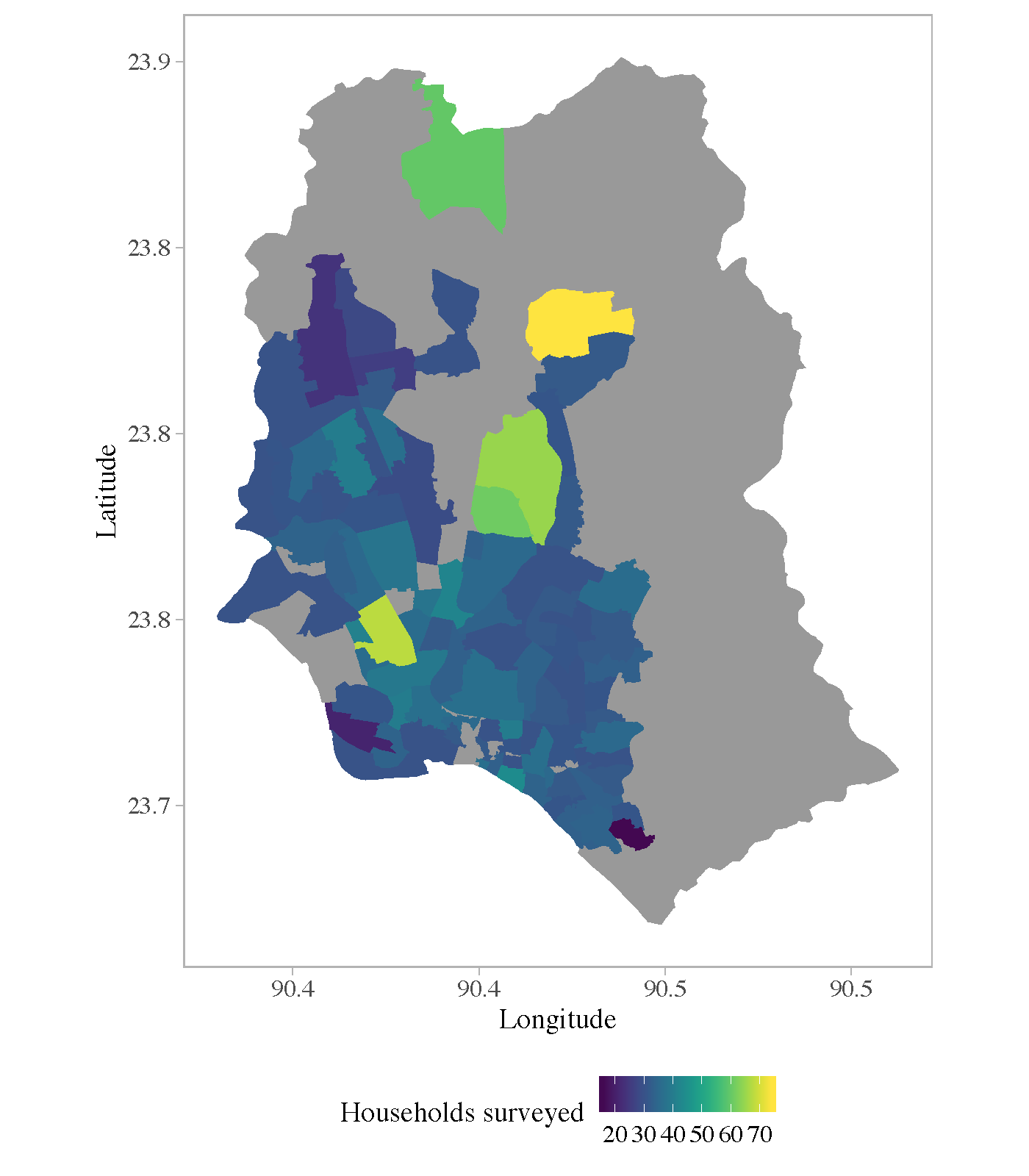

Supplement: S1 Fig — The boundaries of the smallest administrative units (unions) are shown in white. Grey indicates locations that were not part of the survey. (TIFF) [file pntd.0009106.s001.tiff]

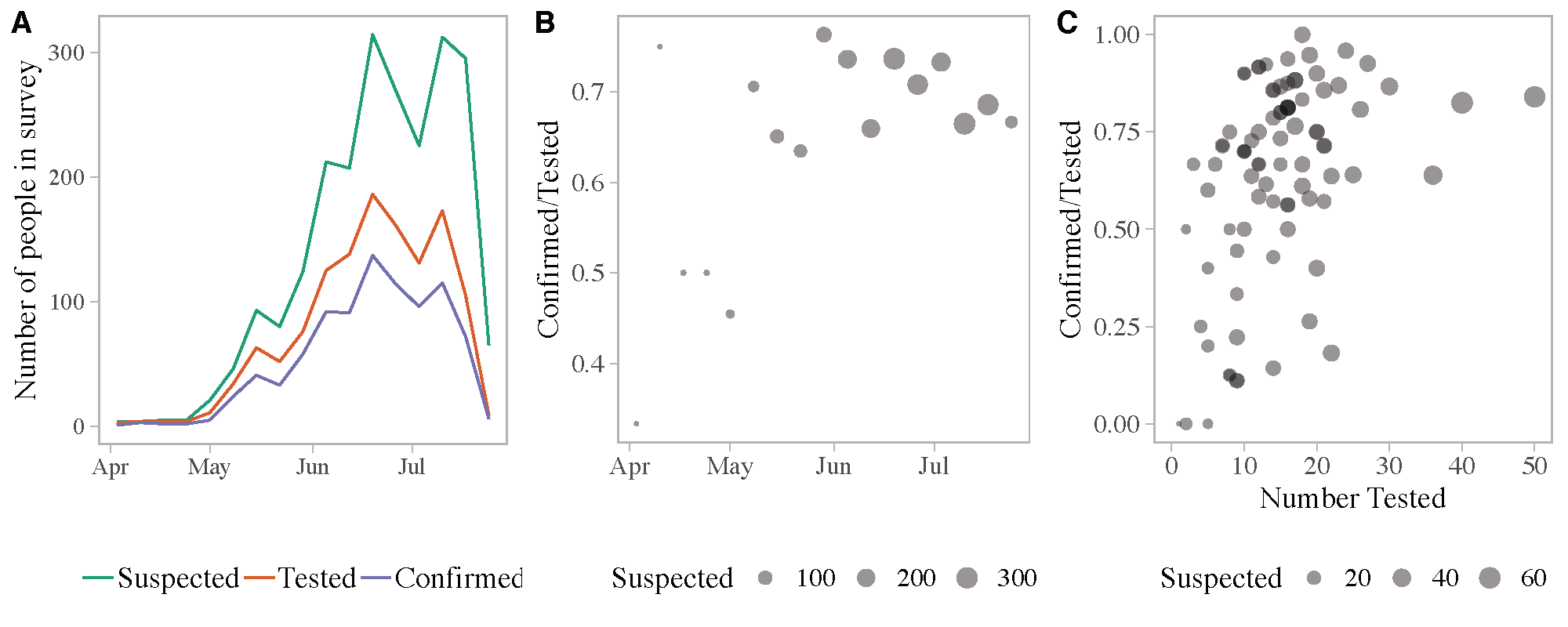

Supplement: S2 Fig — (A) Weekly number of suspected (both tested and non tested) individuals, tested individuals, and confirmed cases in the survey. The ratio of confirmed to tested cases by (B) week and (C) location. While there may be true variation in this ratio across time and space, much of the sample variation appears to be driven by the small number tested in certain weeks or locations. (TIFF) [file pntd.0009106.s002.tiff]

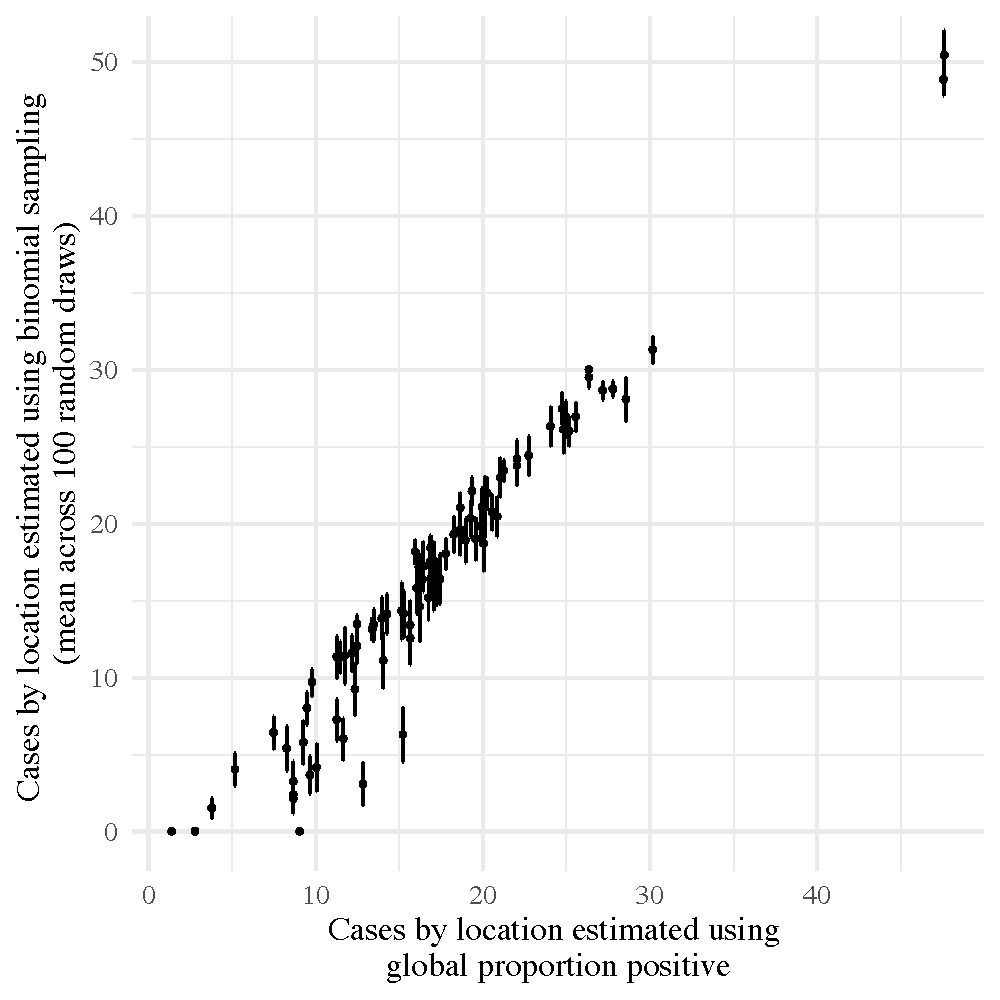

Supplement: S3 Fig — X-axis shows the cases in each location estimated using the global sample ratio of confirmed cases to the number tested. Y-axis shows the cases estimated by drawing from a binomial distribution with size (number of trials) given by the number of untested suspected cases and the probability of a positive case given by the local ratio of confirmed cases to the number tested. The mean and standard deviation (error bars) across 100 random draws are shown here. (TIFF) [file pntd.0009106.s003.tiff]

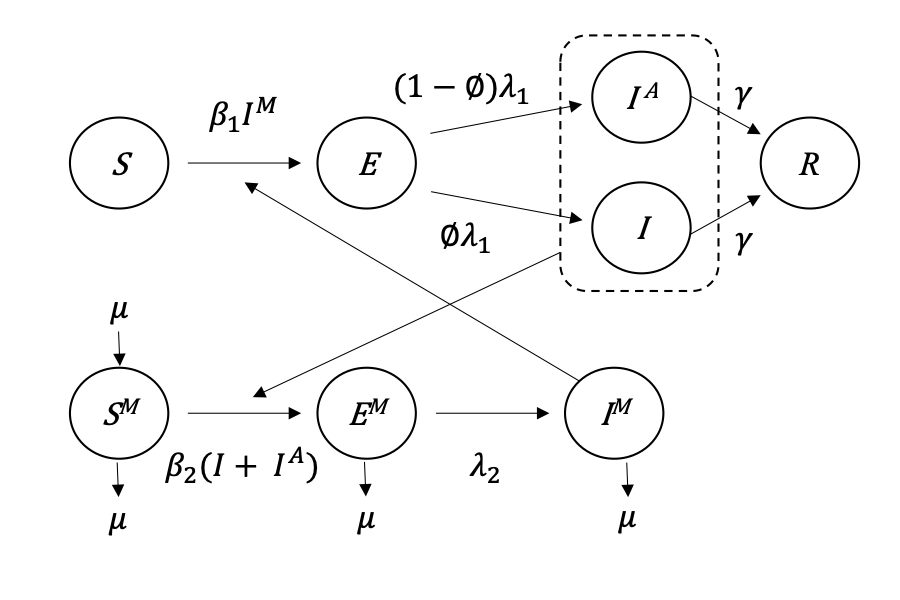

Supplement: S4 Fig — (TIFF) [file pntd.0009106.s004.tiff]

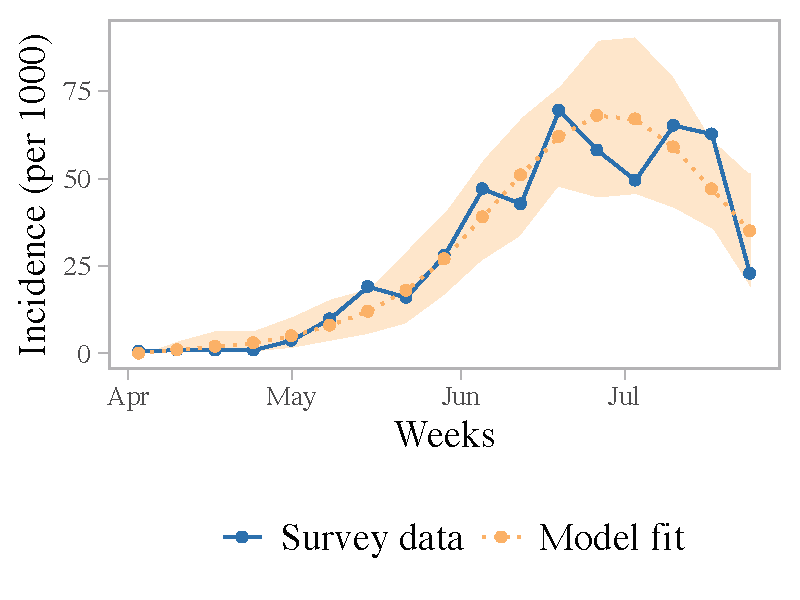

Supplement: S5 Fig — The blue line shows the observed incidence from the survey. The orange dotted line shows the best-fit model. The model was fit to observed data assuming a measurement error structure i.e. we maximize the likelihood that the simulated incidence is drawn from a Poisson distribution with mean given by the observed data. The shaded region shows the full range of incidence for 100 simulations (using the best-fit parameters) and assuming a Poisson error structure. (TIFF) [file pntd.0009106.s005.tiff]

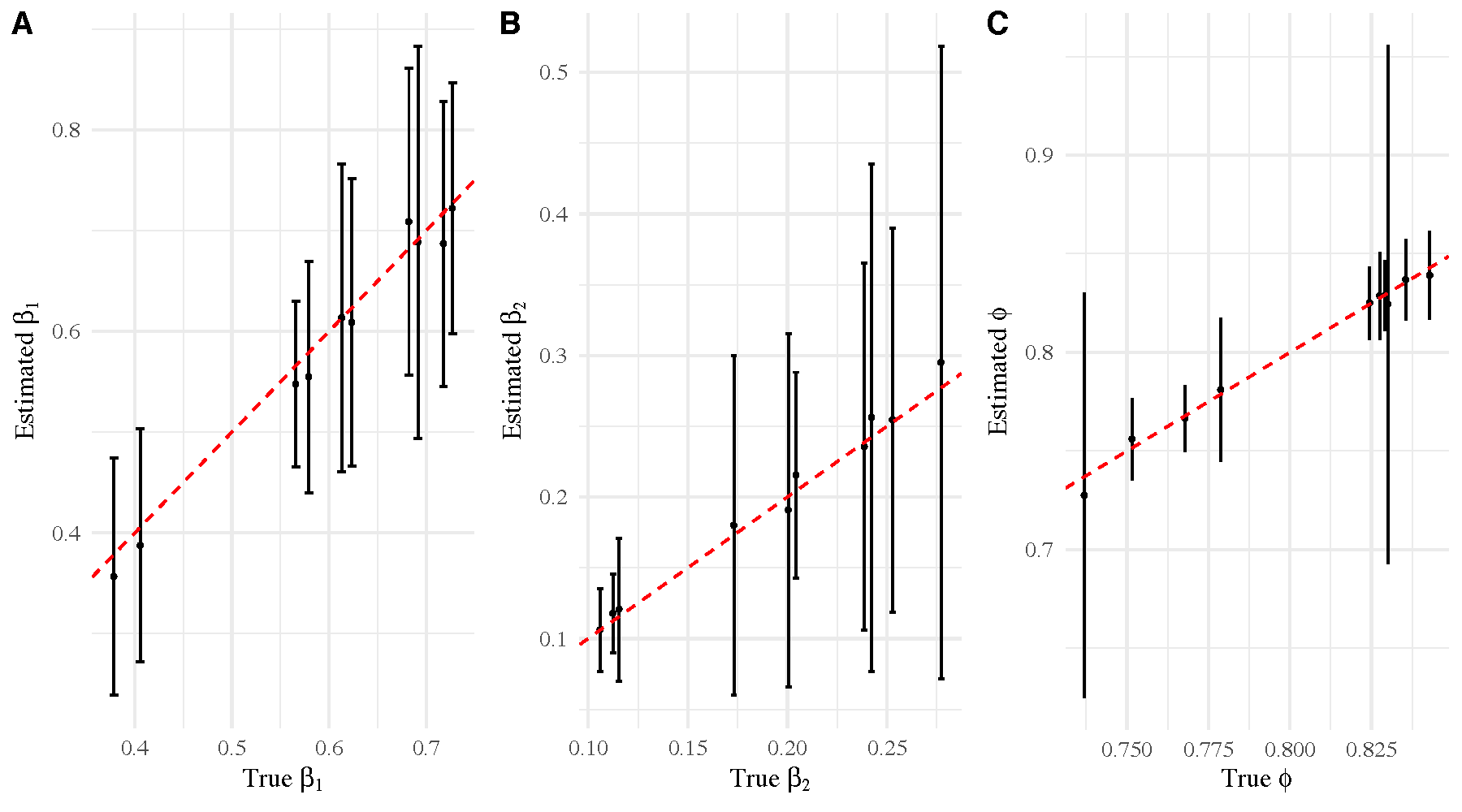

Supplement: S6 Fig — Parameter estimates from fitting the model to simulated data. The simulated data was generated for ten sets of parameter values; true parameter values were randomly drawn from a uniform distribution with minimum and maximum values defined by the 95% CI for each parameter (from the main model fit). For each set of parameter values, 100 incidence trajectories were simulated with a Poisson observational error structure. Median estimates from model fitting and standard deviation across the 100 simulations (error bars) are shown here. The red dashed line indicates the x = y line. In general, the standard deviation is smaller when the peak simulated incidence occurs earlier in the observation window i.e. more of the outbreak is observed. (TIFF) [file pntd.0009106.s006.tiff]

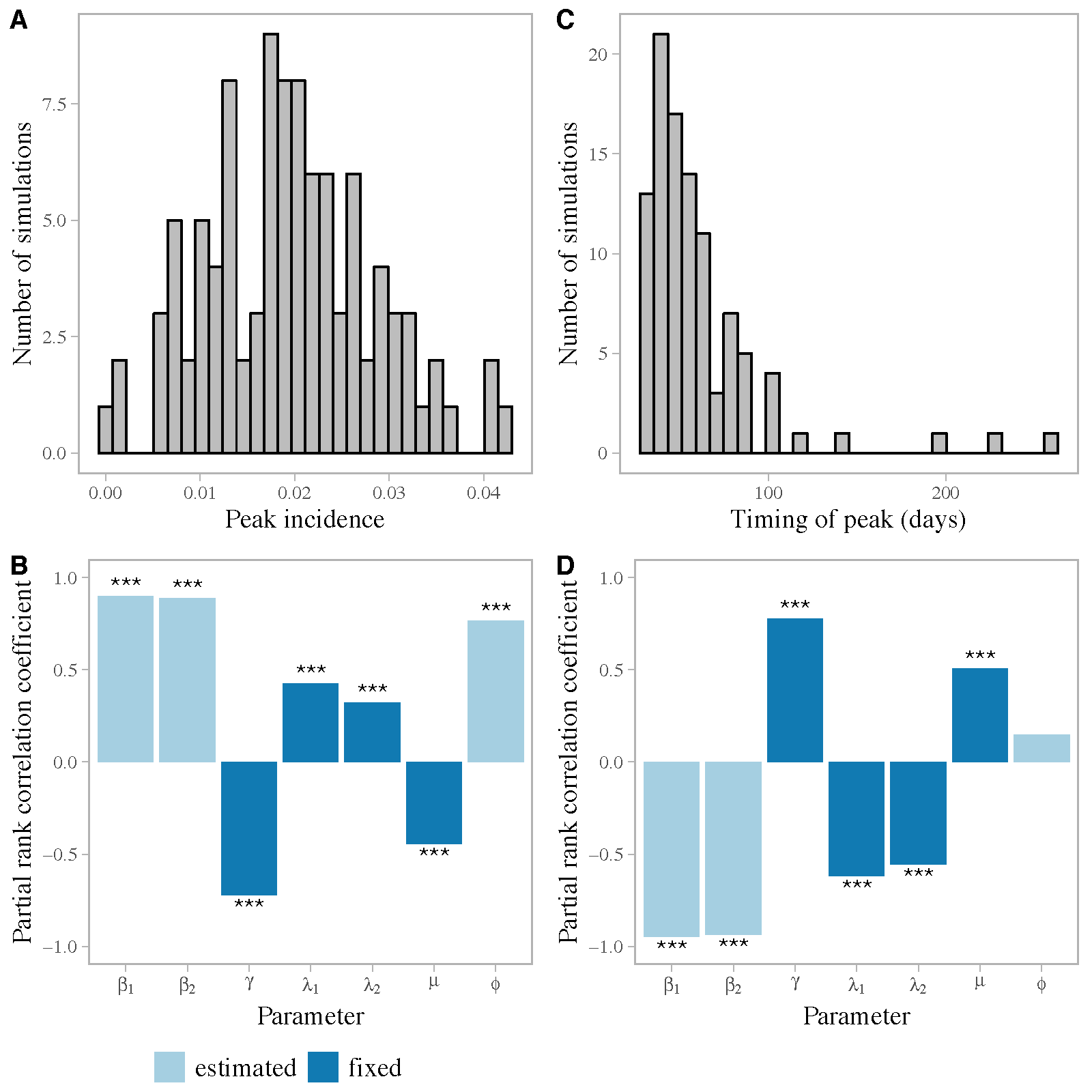

Supplement: S7 Fig — Histogram of model output for (A) peak incidence and (C) the timing of the peak (in days from the start of the outbreak). Parameters were sampled via Latin Hypercube Sampling, and models were simulated using 100 parameter combinations. Partial rank correlation analysis for (B) peak incidence and (D) timing of the peak, to assess sensitivity of model output to each parameter. The height of the bar indicates the correlation of the model output with the given parameter, holding all else equal. Positive values indicate a positive change in the model output in response to an increase in the parameter, while negative values indicate a negative change. Parameters with greater influence have large absolute values of the correlation coefficient. *** indicates p < 0.05. (TIFF) [file pntd.0009106.s007.tiff]

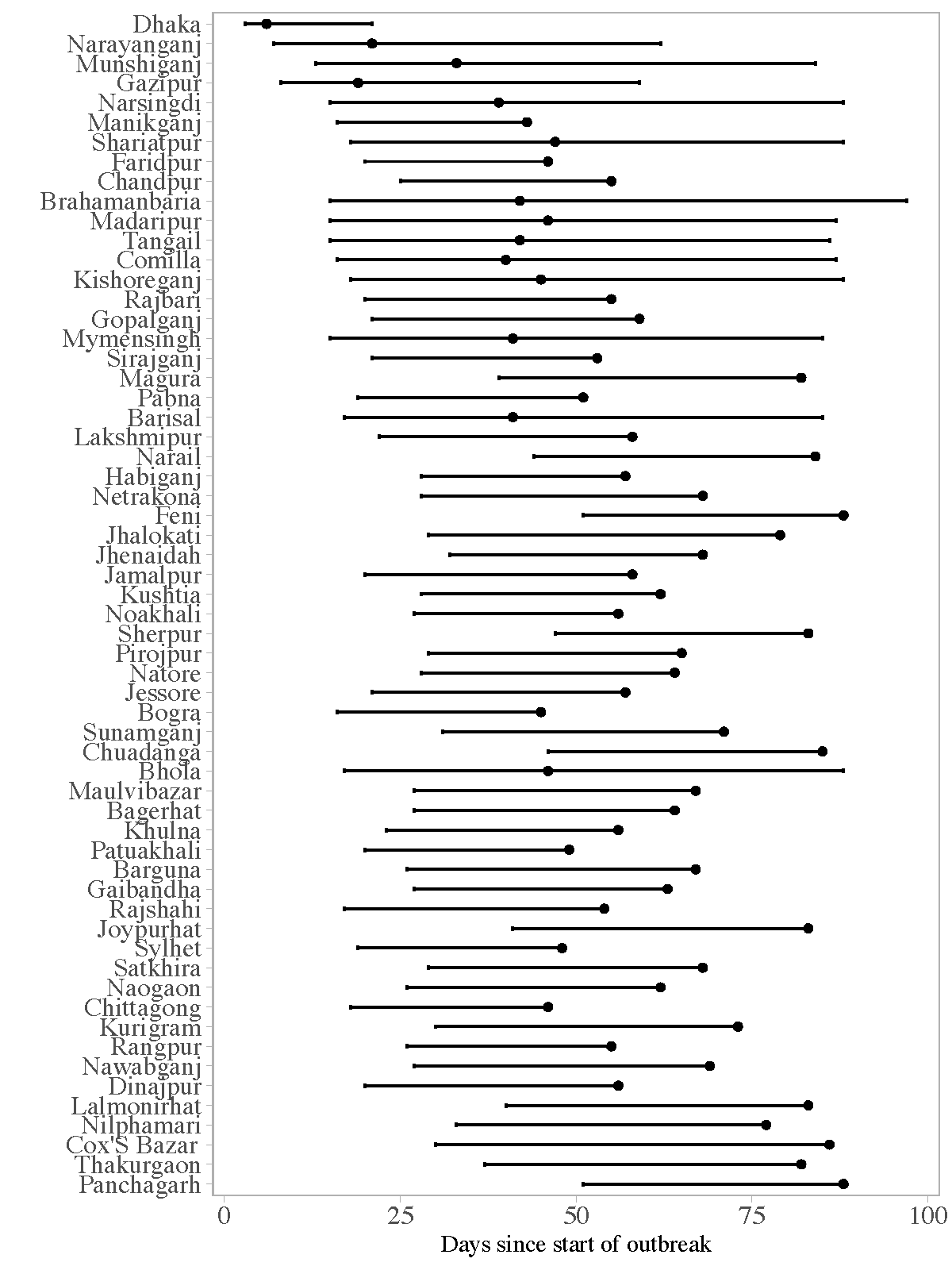

Supplement: S8 Fig — Introduction is defined here as the importation of at least ten cases. For each district, the lowest value (earliest introduction) represents peff = 0.5 and the highest value (latest introduction) represents peff = 0.01. The dots represent the peff = 0.1 scenario, which is used in the main results. For some districts, no importations were predicted with peff = 0.01; for these districts the highest value represents peff = 0.1. (TIFF) [file pntd.0009106.s008.tiff]

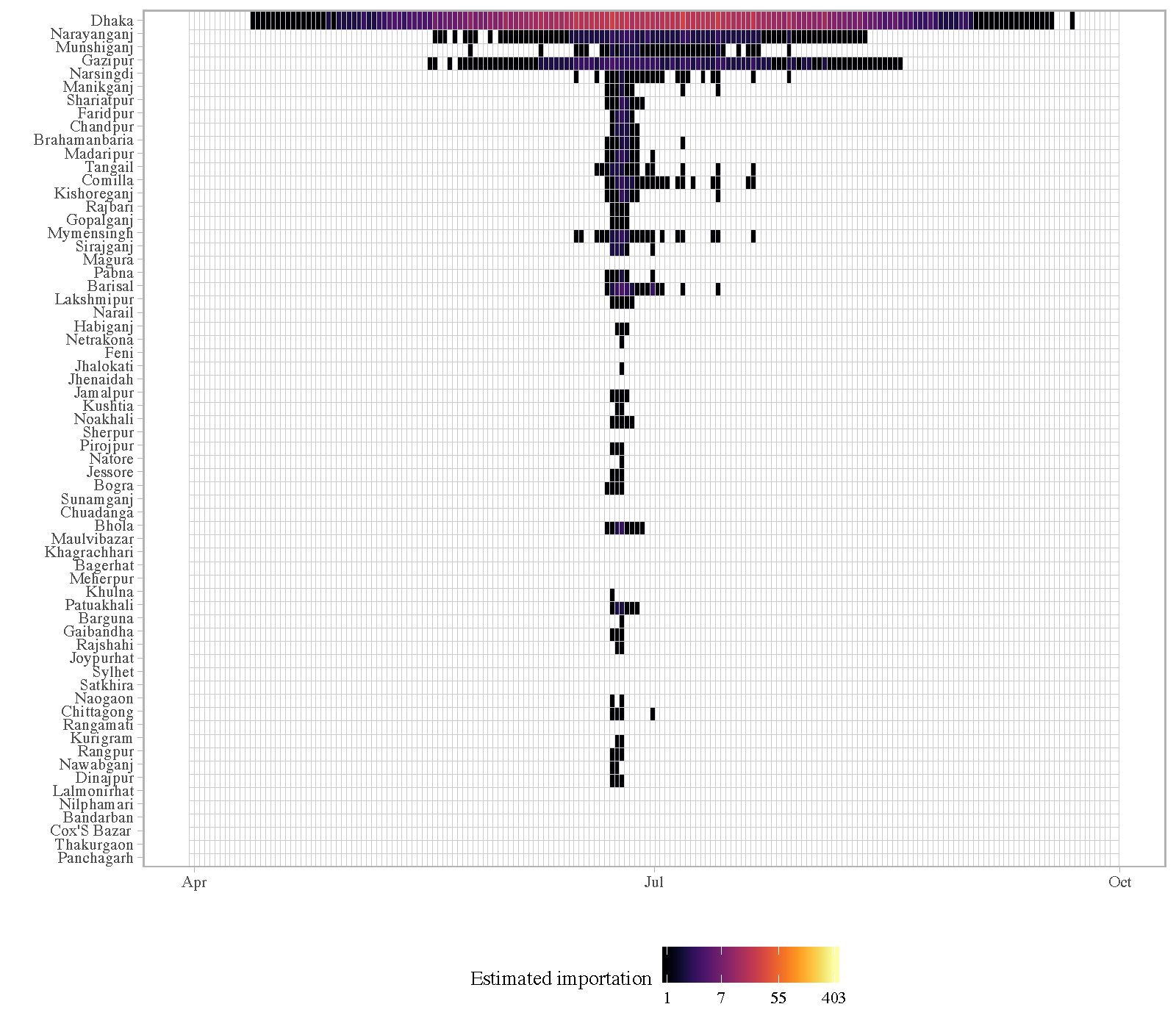

Supplement: S9 Fig — Each row represents a district; districts are arranged from top to bottom by distance from Dhaka in ascending order. (TIFF) [file pntd.0009106.s009.tiff]

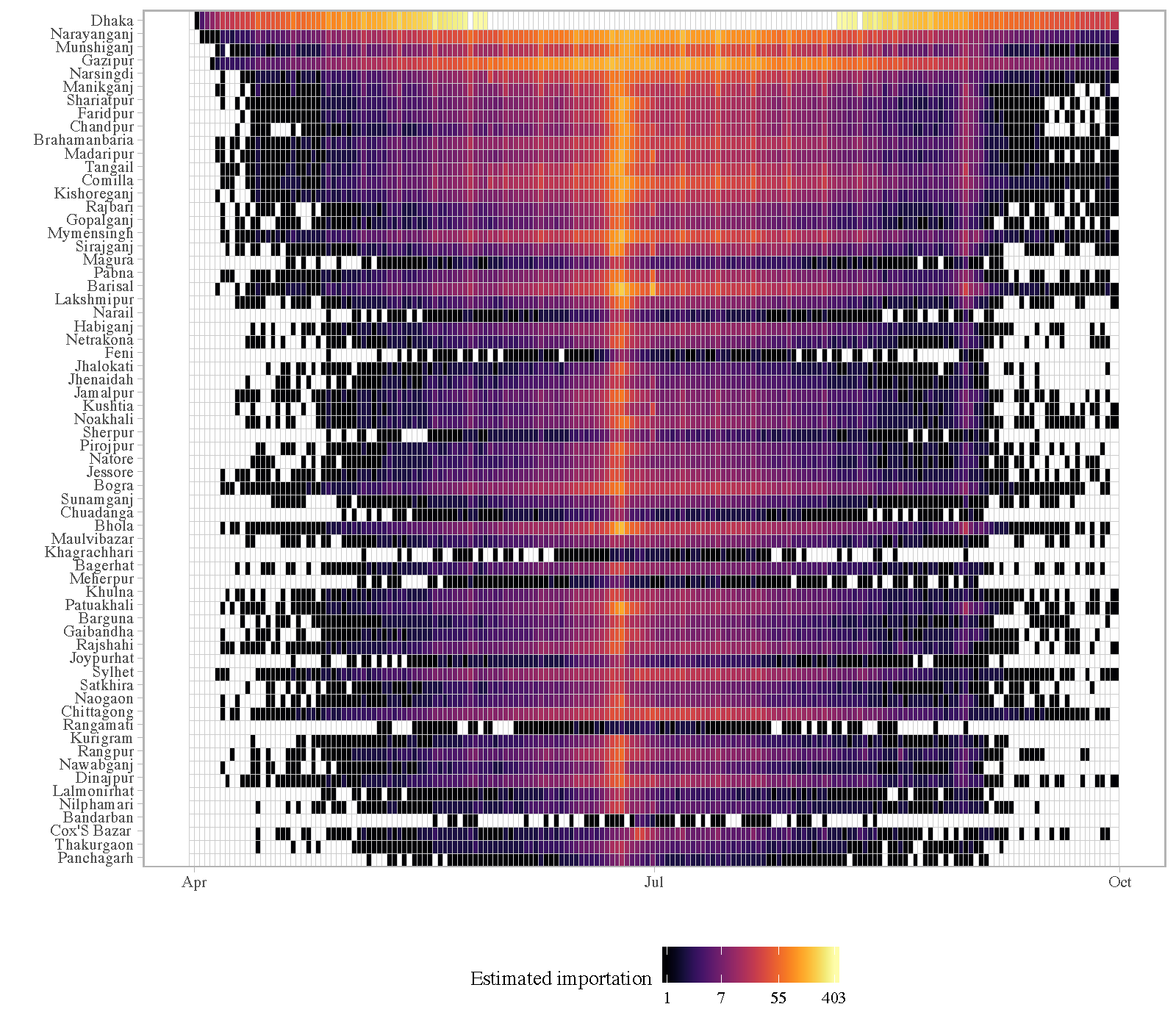

Supplement: S10 Fig — Each row represents a district; districts are arranged from top to bottom by distance from Dhaka in ascending order. (TIFF) [file pntd.0009106.s010.tiff]

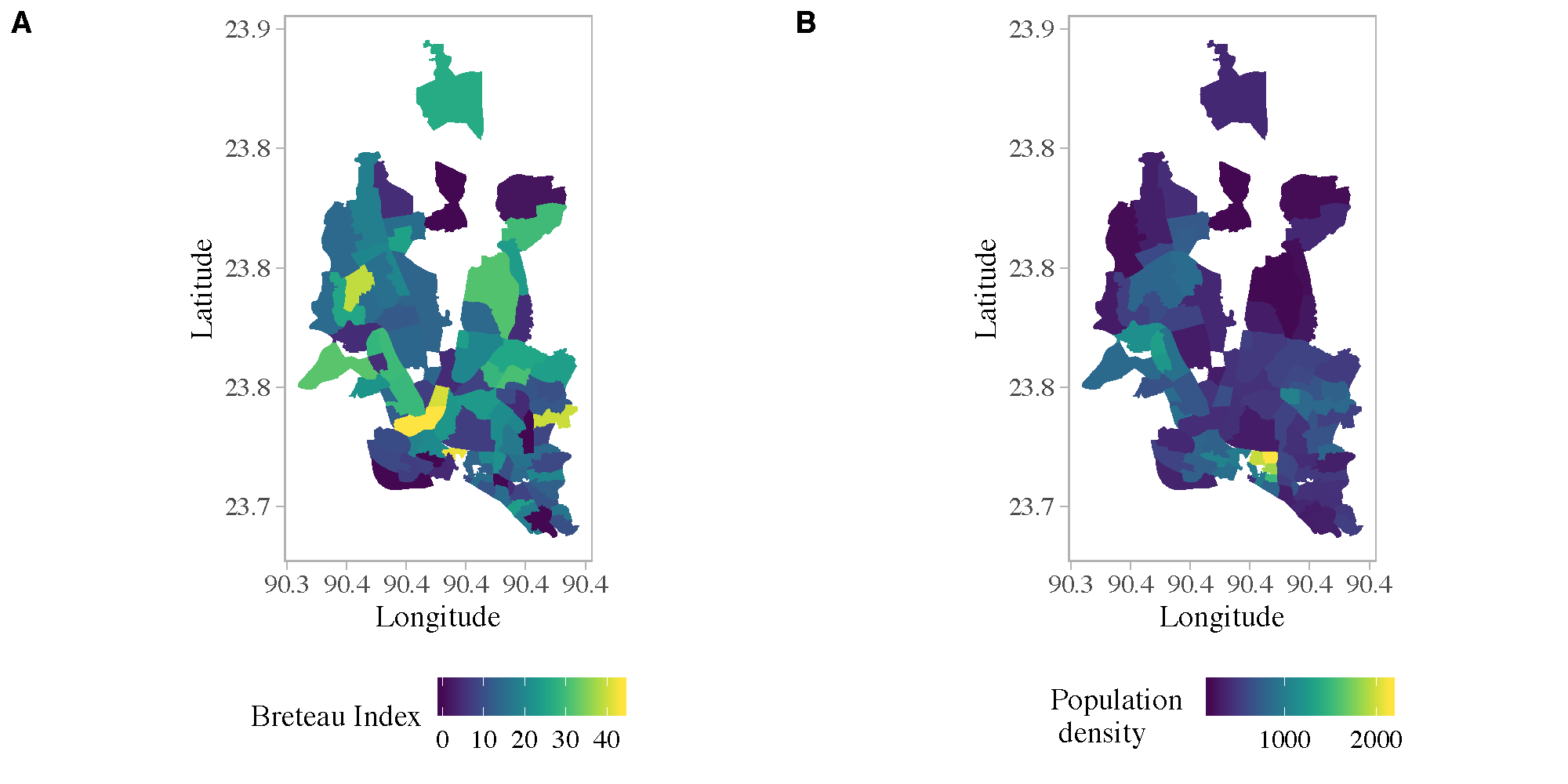

Supplement: S11 Fig — (TIFF) [file pntd.0009106.s011.tiff]

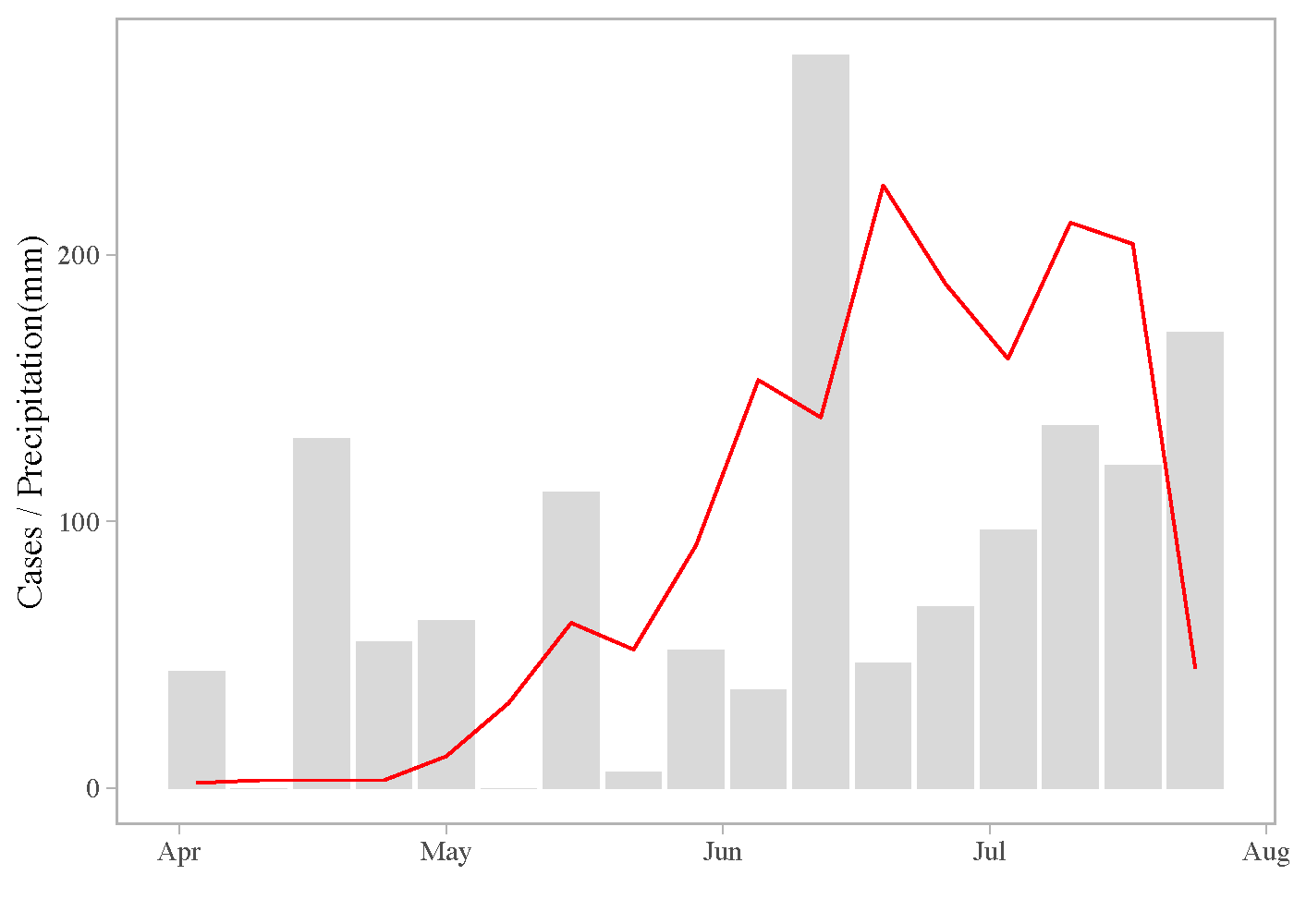

Supplement: S12 Fig — (TIFF) [file pntd.0009106.s012.tiff]

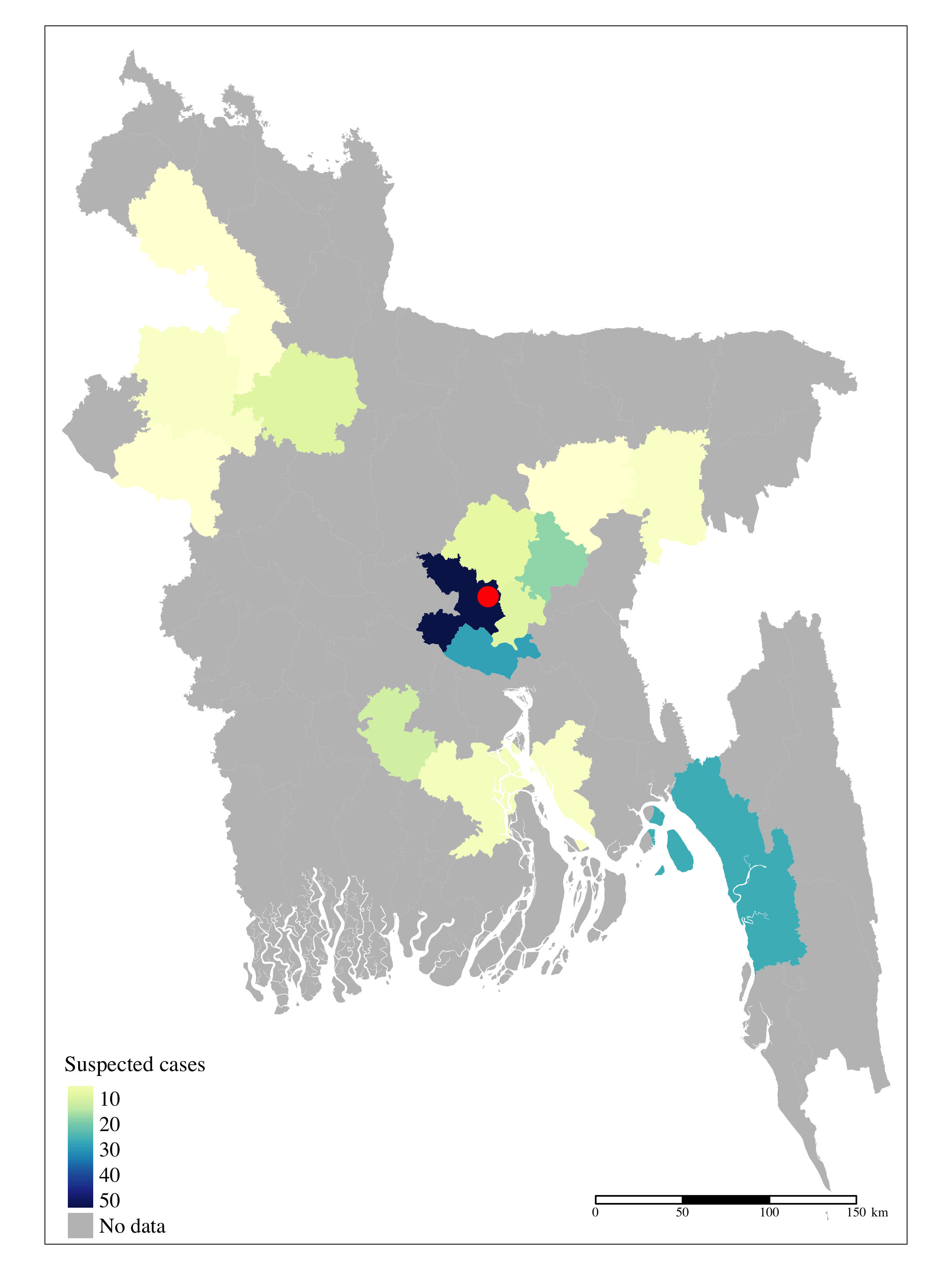

Supplement: S13 Fig — The red dot shows the location of Dhaka city. Cases in Dhaka district (highest number reported) are for locations in Dhaka district outside of the city limits. (TIFF) [file pntd.0009106.s013.tiff]
