# Supplementary material for: Megacities as drivers of national outbreaks: The 2017 chikungunya outbreak in Dhaka, Bangladesh
Source: PLoS Negl Trop Dis. 2021 Feb 2;15(2):e0009106. doi: 10.1371/journal.pntd.0009106 (PMC7880496; doi:10.1371/journal.pntd.0009106)
Supplement: S1 Table — The median and range of estimates for each of the fitted parameters are shown. The initial proportion susceptible was varied from 0.6 to 1 by increments of 0.05. The range of parameter estimates were obtained by fitting the simulated incidence from the mechanistic model to the observed incidence in the survey, assuming the different values for the initial proportion susceptible. (PDF) [file pntd.0009106.s014.pdf]

Parameter	Median	Range (minimum, maximum)
β_1	0.51	(0.45, 0.56)
ϕ	0.78	(0.62,1)
β_2	0.16	(0.12,0.25)

S1 Table: Sensitivity of parameter estimates to the initial proportion susceptible. The median and range of estimates for each of the fitted parameters are shown. The initial proportion susceptible was varied from 0.6 to 1 by increments of 0.05. The range of parameter estimates were obtained by fitting the simulated incidence from the mechanistic model to the observed incidence in the survey, assuming the different values for the initial proportion susceptible.
