# Supplementary material for: Megacities as drivers of national outbreaks: The 2017 chikungunya outbreak in Dhaka, Bangladesh
Source: PLoS Negl Trop Dis. 2021 Feb 2;15(2):e0009106. doi: 10.1371/journal.pntd.0009106 (PMC7880496; doi:10.1371/journal.pntd.0009106)
Supplement: Attachment — Submitted filename: Response to reviewers comments.pdf [file pntd.0009106.s015.pdf]

Dear Editors,

Please find the revision of our manuscript "***Megacities as drivers of national outbreaks: the 2017 chikhungunya outbreak in Dhaka, Bangladesh***". We would like to thank the reviewers for their comments, which we found helpful and constructive. Below we outline the changes we have made to the manuscript based on these comments. We also provide responses to the more specific questions. We believe these changes have made the paper stronger, and we hope that the reviewers' comments are adequately addressed.

Sincerely,
Ayesha Mahmud and Caroline Buckee

Overall major revisions:

1) Based on the reviewers' suggestions, we have added several additional sensitivity analyses of our model fitting and parameter estimation methods in the Supplementary Material; our main results remain unchanged.
2) We have added clarifying details about the epidemiological data and the mechanistic model in the Methods section.
3) We have added more detail on the limitations of our mobility-based predictions, particularly the lack of data on incidence outside Dhaka.

Response to Reviewers' Comments:

Reviewer #1:
I think the objectives and hypotheses are clearly stated, and the methodology presented is relevant to the question. Nevertheless, as the authors claim that it is important to consider such mobility data in epidemiological model, I think it is required that the authors show what is the model best fit without considering these data. If the model can produce similar results without such mobility data, it would highlight that these data improve model fit, but is not a required component.

Mahmud et al address the interesting question of how holiday travel can be involved in the spread of infectious diseases. Based on the outbreak of Chikungunya in Bangladesh in 2017, the authors have integrated mobile phone data within a mechanistic epidemiological model to estimate the contribution of population mobility into the spread of pathogens.

This is an interesting paper, but I need to be more convinced that data from mobile phone really improve model fit in this case. I'm a bit disappointed to see that only the dynamics in

Dhaka is really considered within the mechanistic model. It would have been much more relevant (I think) to consider a metapopulation model to model the whole spatio-temporal dynamics of this outbreak, moreover regarding the level of details of mobile phone data. This would have been a real novelty in the literature, and the kind of model that these data deserve.

Thanks to the reviewer for raising this important issue. Unfortunately, there is very little data available from outside Dhaka for either the 2017 Chikungunya outbreak or previous outbreaks in Bangladesh. As we note in the manuscript, due to the lack of a surveillance and disease reporting system, only a handful of hospitals outside Dhaka reported cases to the Institute of Epidemiology, Disease Control and Research (IEDCR). Since reporting was not mandatory, there is no information about chikungunya incidence in the majority of districts in Bangladesh, and the reporting hospitals only did so for a few weeks. The little information we do have on timing and locations of places reporting, however, suggests that the outbreak had spread to places far from Dhaka by August. No reports (including news reports) of cases outside Dhaka exist prior to June, suggesting that the outbreak originated in Dhaka.

As the reviewer correctly notes, a complete meta-population model would be ideal for capturing the complete picture of spatio-temporal dynamics. However, in the absence of data on the outbreak outside Dhaka, such a model would be impossible to parametrize. This is especially difficult for vector-borne pathogens since we would have little or no data to inform the mosquito-to-human and human-to-mosquito transmission rates which are likely to vary by location. We agree with the reviewer that such a model will be valuable for understanding the full spatial dynamics and hope that improved surveillance and/or seroprevalence studies outside Dhaka can inform such models in the future.

While we cannot estimate a fully meta-population spatial model, our estimates using the mobile phone data clearly highlights the deviation from a null model (diffusion or gravity model). We predicted a high likelihood of importation into areas with large volumes of travel from Dhaka, particularly during the Eid holidays, which cannot be captured in the absence of mobile phone data. Compared to the naive diffusion model, the model parameterized with mobile phone data showed much greater variation in imported cases across space and time (Figs 4 and 5). Specifically, for some districts that reported suspected cases, such as Bogra and Dinajpur, we predicted a larger number of imported cases than would be expected based on its distance from Dhaka. In the diffusion model importation decayed rapidly with distance, with very few imported cases to locations far from Dhaka. In contrast, the mobility estimates showed high volumes of travel to some locations, such as Dinajpur district (which reported cases), that are far from Dhaka.

In summary, the mobile phone data reveals that actual travel patterns in Bangladesh are significantly different than what would be expected based on a diffusion model (which is

commonly used in the absence of mobility data), particularly during the Eid holidays, and this has important implications for the spread of diseases. We acknowledge the important limitation that the reviewer notes but are unable to address this without data from other parts of the country. We have now added a more detailed discussion of this in the Introduction, Results and Discussion sections.

Reviewer #2:

L70 : - the sample should be described more consistently: 3253 Hsld were included. How many individuals had a symptomatic history?
1487 were sampled : what fraction of all cases is it? 1286 had IgM tested : what happened with the 201 other cases?
Why were there some districts of Dhaka not included in the study?

Thanks to the reviewer for raising this issue, and we apologize for this oversight and omitting this important detail. We have now added additional detail to the Materials and Methods section. Of the 3253 households, 77% of individuals (2518) met the clinical definition (fever, rash and joint pain). Of these symptomatic individuals, blood samples were collected from 1487 randomly selected individuals. However, the ICT test was performed on a smaller subset of randomly selected samples (1286) and 201 samples were not tested. This comprises just over 50% of suspected cases.

The wards (unions) at the core of Dhaka city (i.e. most populated) were included in the survey. This included 92 wards and 8 peri-urban areas of Dhaka North and South city corporations. These served as the 100 clusters, with 30 households randomly selected from within each cluster. The area missing within the core of Dhaka city (shown in Figure 1) consists of the airport and the Army cantonment area (neither of which have civilian households).

L114 : the way you impute missing data reduces the heterogeneity in the percentage positive. The way O is computed may lead to values being overly influenced by the results of districts with large C and T. Using a mixed/random model with binomial sampling would be a better approach. This is all the more relevant since the association analysis shows may be anticonservative due to discounting uncertainty in reconstructing O.

Thanks to the reviewer for bringing up this important point. We do see some variation in the proportion of tests that are positive across time and space (Figure S2 B and C). However, we see little variation from June onwards (when a greater number of tests were done). The deviation in the proportion positive from the sample mean is also greatest for places with low numbers tested. While there may be true variation in this ratio across time and space, much of the sample

variation appears to be driven by the small number tested in certain weeks or locations. Thus, we chose to use the overall global proportion positive to impute the total number of symptomatic cases for each location.

Based on the reviewer's suggestion, however, we also estimated O_i (number of cases in location i) using the observed local proportion positive (C_i / T_i) for each location rather than the global proportion across the sample. We did so by sampling from a binomial distribution with size (number of trials) given by S_i (reported symptoms but not tested) and the probability of a positive case given by the local proportion positive (C_i / T_i). We repeated the sampling 100 times and examined the variability in the estimated O_i for each location. We find that the standard deviation across the 100 samples is small compared to the overall variation in O_i across locations. Furthermore, the mean value of O_i across the 100 samples is very close to our estimated O_i using the global proportion positive (see Figure below). We have now included this as a sensitivity analysis in the Supplementary Material (Figure S3).

Finally, an advantage of using a regression-based approach for the association analysis is that measurement error in the dependent variable, O_i, should not affect the estimated associations with the independent variables. The estimated coefficients would still be an unbiased estimate of the underlying values; however, measurement error in the dependent variable will reduce the precision of these estimates. The standard errors of our coefficient estimates will increase due to the increase in the standard error of the regression (owing to the added "noise" in the dependent variable, O_i). As such, our confidence intervals will be wider.

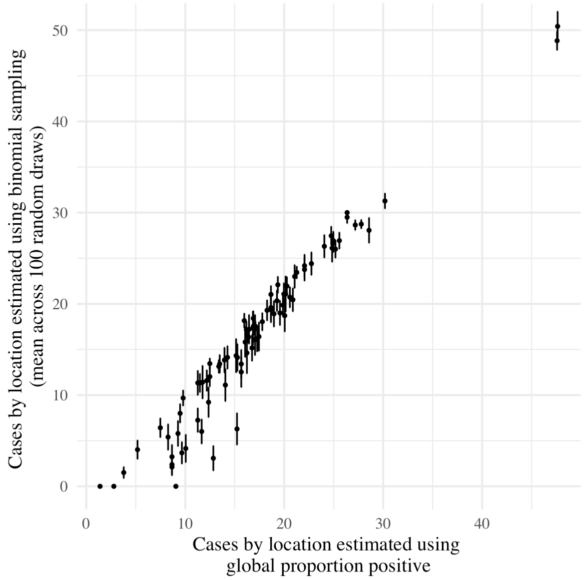

Figure S3: Cases in each location estimated using the global sample ratio of confirmed cases to the number tested versus cases estimated by drawing from a binomial distribution with size (number of trials) given by the number of untested suspected cases and the probability of a

positive case given by the local ratio of confirmed cases to the number tested. The mean and standard deviation (error bars) across 100 random draws are shown here.

L113 : How long does it take for igM to be positive relative to infection & sampling? should we expect unerestimation here?

Thanks to the reviewer for raising this issue. As noted in the manuscript, IgM antibodies for CHIKV can be detected 3 to 4 days after clinical onset, and remain detectable for 1 to 3 months post-infection. Due to the retrospective nature of the survey, and the fact that the majority of those reporting symptoms did so more than a week before the survey, we expect the under-estimation due to the delay to be minimal (and only affecting those who reported symptoms in the final week of the survey). We have now added a sentence in the Material and Methods section to acknowledge this potential source of bias.

L179 : Definition of R0. Please indicate that this is the "human-specific" R0, i.e. human to human. The literature is not totally consistent regarding the definition of R0. However, the next generation method should yield the square root of the reported quantity, i.e human -> moquito (or mosquito -> human) This is described in Roberts M.G., Heesterbeek J.A.P. A new method for estimating the effort required to control an infectious disease. Proceedings of the Royal Society of London B: Biological Sciences. 2003;270(1522):1359–1364 and in van der driessche papers.

Thanks to the reviewer for bringing up this very important point. We have now added text to the Materials and Methods section to clarify this. We now refer to the human-to-human R_0 as R_0^H.

L205: modelling incidence as a gaussian variable in the fit of the model is questionable. Poisson or NegBin is far more reasonable, as you did in the first part of the analysis, because variance is unlikely to be constant with incidence. Does it make a difference if you fit the model using a Poisson ?
Furthermore it seems that this is what you do in the computation of the confidence intervals. Is there a reason you use different models for analysing the data and performing parametric bootstrap?
In theory, parametric bootstrap is valid if your model is well specified. This seems unlikely if it is not the same for simulation and estimation. Another possibility would be to estimate parameter variance from Fisher's information matrix.

Thanks to the reviewer for these very useful suggestions. In order to estimate the model parameters, we fit the simulated model trajectory to the observed outbreak data, assuming that the residuals are normally distributed (equivalent to a least-squares estimation method). The 95% confidence intervals for the parameter estimates are then constructed using a set of realizations of

the best-fit curve assuming an observational error structure that follows a Poisson distribution. This method for model fitting and constructing nominal 95% confidence intervals has been used previously for estimating the reproduction number of various pathogens by fitting to case notification data (for example, Chowell et al. (2006), Chowell et al. (2007) and Viboud et al. (2016)). As has been noted in previous work, "these confidence intervals should be interpreted as containing 95% of future estimates when the same assumptions are made and the only noise source is observation error". And as the reviewer notes, the parametric bootstrap method is only valid if the underlying assumptions hold. We have now included a sentence in the Materials and methods section to acknowledge this important limitation of our method.

As per the reviewer's suggestion, we also fit simulated model trajectories to the observed outbreak data by assuming a measurement error structure i.e. we maximize the likelihood that the simulated number of infected is drawn from a Poisson distribution with mean given by the observed data. Our results are qualitatively very similar (see Figure below), and we have now included this additional analysis in the Supplementary Material.

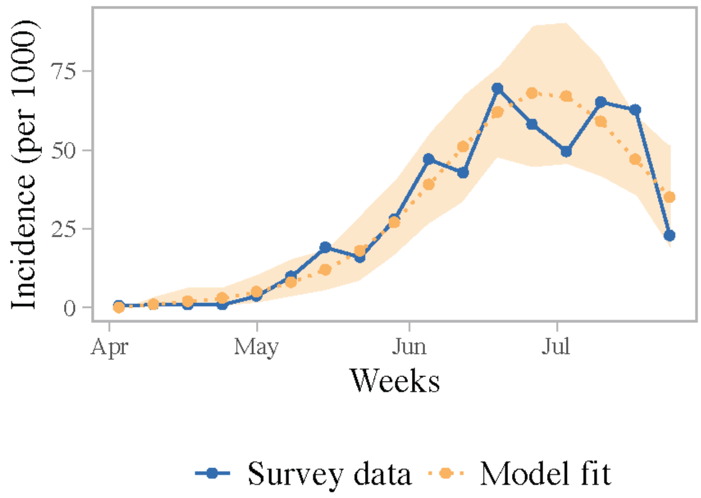

Figure S5: Observed and fitted weekly incidence. The blue line shows the observed incidence from the survey. The orange dotted line shows the best-fit model. The model was fit to observed data assuming a measurement error structure i.e. we maximize the likelihood that the simulated incidence is drawn from a Poisson distribution with mean given by the observed data. The shaded region shows the full range of incidence for 100 simulations (using the best-fit parameters) and assuming a Poisson error structure.

L209 : If you used the Nelder Mead algorithm, why did you require LHS sampling?

To account for the potential sensitivity of the model fitting algorithm to initial conditions, the best-fitting parameters were obtained by a 100-fold repeat of our fitting procedure with different initial conditions (for the parameters to be estimated) randomly drawn from appropriate

parameter ranges. We used LHS sampling to explore this parameter space to specify the set of initial conditions for each repeat of the fitting algorithm. We find that the maximum-likelihood parameter estimates were stable across a range of initial starting conditions.

L211: I'm not convinced that both beta1 and beta2 can be estimated with the data that you have.
You certainly have information on the product beta1 * beta2 that defines the growth rate of the epidemic curve as seen in the R0 formula. Can you provide simulation data showing that joint estimation is indeed possible with the data you have?

Thanks to the reviewer for this great suggestion. We have now included a parameter identifiability analysis to the Supplementary Material. Using simulations, we find that joint estimation of β_1 and β_2 is possible when there is little or no noise in the data. Our estimation method is able to recover the parameter values used for the simulation. However, as we add observational error we see variability in the parameter estimates (which we aim to capture with our bootstrapped CIs). Even with observational error, we are generally still able to jointly estimate β_1 and β_2 (see Figure below). We have now included this in our Supplementary Material.

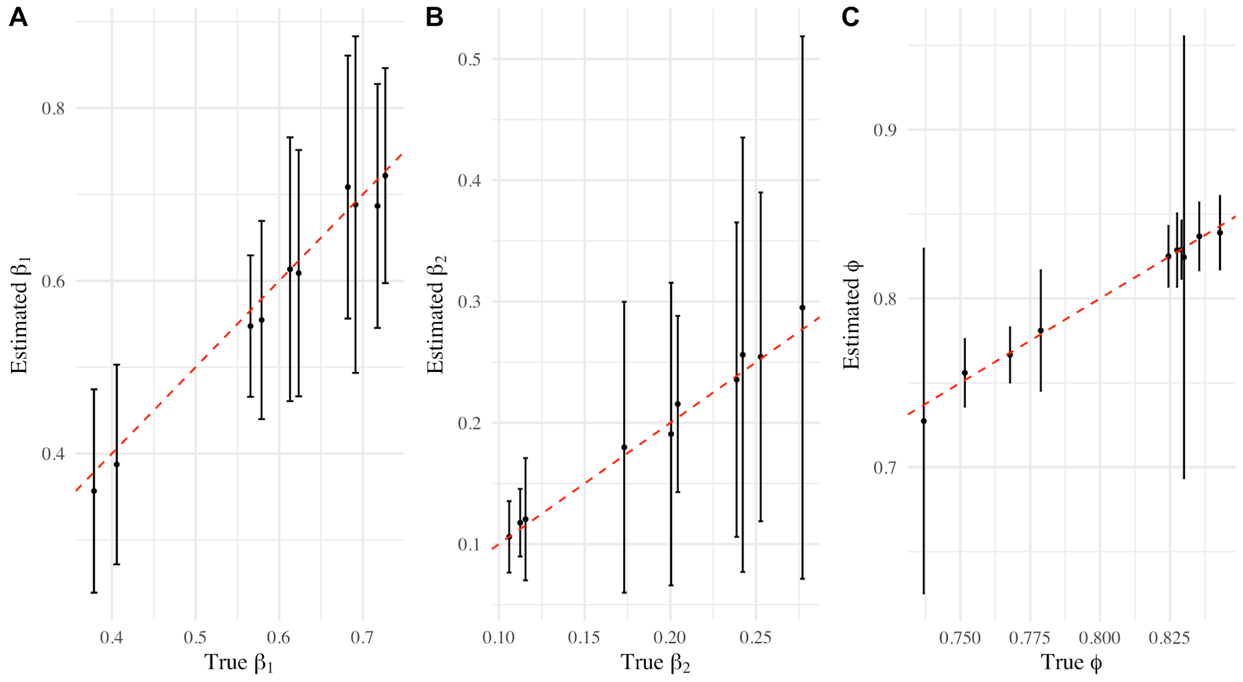

Figure S6: Parameter identifiability analysis. Parameter estimates from fitting model to simulated data. The simulated data was generated for ten sets of parameter values; "true" parameter values were randomly drawn from a uniform distribution with minimum and maximum values defined by the 95% CI for each parameter (from the main model fit). For each set of parameter values, 100 incidence trajectories were simulated with a Poisson observational

error structure. Median estimates from model fitting and standard deviation across the 100 simulations (error bars) are shown here. The red dashed line indicates the x=y line. In general, the standard deviation is smaller when the peak simulated incidence occurs earlier in the observation window i.e. more of the outbreak is observed.

You further report that "The majority of the starting values converged to the same maximum-likelihood parameter estimate for 1 (median = 0.51, 25th percentile = 0.51, 75th percentile = 0.52), 2 (median = 0.16, 25th percentile = 0.16, 75th percentile = 0.16) and (median = 0.78, 25th percentile = 0.78, 75th 215 percentile = 0.78).". Does this mean your likelihood present several maxima?

We have now clarified this in the Material and Methods section. To account for the potential sensitivity of the model fitting algorithm to initial conditions, the best-fitting parameters were obtained by a 100-fold repeat of our fitting procedure with different initial conditions (for the parameters to be estimated) randomly drawn from appropriate parameter ranges. We used LHS sampling to explore this parameter space to specify the set of initial conditions for each repeat of the fitting algorithm. We find that the maximum-likelihood parameter estimates were stable across a range of initial starting conditions.

L213: regarding the rates, are the ranges OK? in normalized models with mosquito-borne transmission, we have beta1 m = beta2 where m is the number of mosquito per human. Furthermore, the betas correspond with frequency of bites, which is controlled by the gonotrophic cycle in mosquitoes.
The mosquito to human rate is beta1 estimated at 0.51. Does this mean that an infected mosquito bites someone every 2 days? this is somewhat short given the gonotrophic cycle for Aedes, even in Dhaka, should be more in the 3-4 days range. The human to mosquito means that an infected human is bitten every 1/0.16 days. With 4 days being infectious, this amounts to less than one bite on average during the infectious period. Some discussion of these numbers is necessary, if really they can be estimated.

Thanks to the reviewer for raising this issue. In our model, β_1 is the rate at which mosquitoes infect humans, and is equivalent to *abm* in the Ross-Macdonald style models, where *a* is biting rate, *b* is the parasite transmissibility to humans and *m* is the ratio of mosquitoes to humans. Thus, following the convention of the Ross-Macdonald model, the rate at which mosquitoes infect humans (β_1) is dependent on *m*, but β_2 is independent of *m*. Our estimated β_1 of 0.51 is equivalent to the number of mosquito bites per human per day resulting in transmission (accounting for imperfect transmission of the pathogen). Our estimates for β_1 and β_2 are very similar to estimates from an outbreak in Reunion Island using the same model (Yakob and

Clements, 2013). We have now added a more detailed discussion of these parameters in the Results section.

For the whole part on cell phone versus gravity model, little is shown to quantify that the mobility data actually does a better job to describe the data in bangladesh and to evidence that Dakha and mobility were the driver of the epidemic as indicated in the title.
Figure 5 shows the change in importation risk, but both quantities seems rather parallel. Is it possible to test that the places at risk are different and better predicted with the phone data? You should try using the date and place of importation to come up with some evidence that cellphone data is more of interest than the gravity model, otherwise this remains hypothetical. Some more elaborated analysis is required for this part if you want to conclude on the use of cellphone data rather than another model of mobility in this particular instance.

Thanks to the reviewer for raising this important limitation. Unfortunately, there is very little data available from outside Dhaka for either the 2017 chikungunya outbreak or previous outbreaks in Bangladesh. As we note in the manuscript, due to the lack of a surveillance and disease reporting system, only a handful of hospitals outside Dhaka reported cases to the Institute of Epidemiology, Disease Control and Research (IEDCR). Since reporting was not mandatory, there is no information about chikungunya incidence in the majority of districts in Bangladesh, and the reporting hospitals only did so for a few weeks. The little information we do have on timing and locations of places reporting, however, suggests that the outbreak had spread to places far from Dhaka by August. No reports (including news reports) of cases outside Dhaka exist prior to June, suggesting that the outbreak originated in Dhaka.

A complete meta-population model would be ideal for capturing the complete picture of spatio-temporal dynamics. However, in the absence of data on the outbreak outside Dhaka, such a model would be impossible to parametrize. This is especially difficult for vector-borne pathogens since we would have little or no data to inform the mosquito-to-human and human-to-mosquito transmission rates which are likely to vary by location.

While we cannot estimate a fully meta-population spatial model, our estimates using the mobile phone data clearly highlights the deviation from a null model (diffusion or gravity model). We predicted a high likelihood of importation into areas with large volumes of travel from Dhaka, particularly during the Eid holidays, which cannot be captured in the absence of mobile phone data. Compared to the naive diffusion model, the model parameterized with mobile phone data showed much greater variation in imported cases across space and time (Figs 4 and 5). Specifically, for some districts that reported suspected cases, such as Bogra and Dinajpur, we predicted a larger number of imported cases than would be expected based on its distance from Dhaka. In the diffusion model importation decayed rapidly with distance, with very few imported cases to locations far from Dhaka. In contrast, the mobility estimates showed high

volumes of travel to some locations, such as Dinajpur district (which reported cases), that are far from Dhaka.

In summary, the mobile phone data reveals that actual travel patterns in Bangladesh are significantly different than what would be expected based on a diffusion model (which is commonly used in the absence of mobility data), particularly during the Eid holidays, and this has important implications for the spread of diseases. We acknowledge the important limitation that both reviewers have noted, but are unable to address this without data from other parts of the country. We have now added a more detailed discussion of this limitation in the Introduction, Results and Discussion sections.

You report that up to 50% of the population was infected during the epidemic. However, you mention that 3 chikungunya epidemics already occured in the last 10 years. Given the attack rate that you estimated here, almost 50%, one would have expected that the percentage of the population that is actually susceptible would be far less than what you hypothesize. This should be discussed.

Thanks to the reviewer for this comment. We have now added additional clarification to the Materials and Methods section. Two of the three outbreaks occurred in rural parts of the country outside Dhaka and affected a much smaller population. In 2011 an outbreak occurred in Dhaka district, although the majority of cases were reported in the Dohar region outside the city. Unfortunately, very little is known about seroprevalence in Dhaka prior to the 2017 outbreak. In the survey population, about 5% of individuals had been infected with CHIKV prior to 2017 (IgG positive and IgM negative), although the actual proportion is likely to be higher since only individuals who reported having recent symptoms were tested. We varied the initial proportion susceptible in our sensitivity analyses, and found that the parameter estimates were stable across a range of values for the initial proportion susceptible (Table S1 Table). Our estimated attack rate is consistent with estimates from chikungunya outbreaks in other settings, and the proportion seropositive estimated from our survey was similar to independent estimates from other studies from Dhaka using a convenience sample (Hossain et al. 2017) and hospital data (Haroon et al. 2018).

all the data must be made available, in some useful aggregate form.

Unfortunately, due to regulations from the Bangladesh Telecommunication Regulatory Commission and the Ministry of Health we are unable to make the data publicly available. We will, however, provide aggregated data to researchers upon request with appropriate data sharing agreements. All code will be made publicly available via GitHub.

References cited:

Chowell G, Diaz-Due˜nas P, Miller JC, Alcazar-Velazco A, Hyman JM, Fenimore PW, et al. Estimation of the reproduction number of dengue fever from spatial epidemic data. Mathematical Biosciences. 2007;208(2):571–589.doi:10.1016/j.mbs.2006.11.011.

Chowell G, Nishiura H, Bettencourt LMA. Comparative estimation of the reproduction number for pandemic influenza from daily case notification data. Journal of the Royal Society Interface. 2007;4(12):154–166. doi:10.1098/rsif.2006.0161.

Viboud C, Simonsen L, Chowell G. A generalized-growth model to characterize the early ascending phase of infectious disease outbreaks. Epidemics. 2016;15:27–37. doi:10.1016/j.epidem.2016.01.002.

Yakob L, Clements ACA. A Mathematical Model of Chikungunya Dynamics and Control: The Major Epidemic on R´eunion Island. PLoS ONE. 2013;8(3):1–6. doi:10.1371/journal.pone.0057448.

Hossain MS, Hasan MM, Islam MS, Islam S, Mozaffor M, Khan MAS, et al. Chikungunya outbreak (2017) in Bangladesh: Clinical profile, economic impact and quality of life during the acute phase of the disease. PLoS Neglected Tropical Diseases. 2018;12(6):1–16. doi:10.1371/journal.pntd.0006561.

Haroon ORM, Patwary MMH, Faruk SM, et al. Seroprevalence of Chikungunya during Outbreak in Dhaka, Bangladesh in 2017. Journal of Virology & Antiviral Research. 2018;07(02):2–5. doi:10.4172/2324-8955.1000184.
